# Analytic and clinical validity of thyroid nodule mutational profiling using droplet digital polymerase chain reaction

**DOI:** 10.1186/s40463-018-0299-2

**Published:** 2018-09-24

**Authors:** Vincent L. Biron, Ashlee Matkin, Morris Kostiuk, Jordana Williams, David W. Cote, Jeffrey Harris, Hadi Seikaly, Daniel A. O’Connell

**Affiliations:** 1 Division of Otolaryngology-Head and Neck Surgery, University of Alberta, 8440-112 st, 1E4 Walter Mackenzie Centre, Edmonton, AB T6G 2B7 Canada; 2 Alberta Head and Neck Centre for Oncology and Reconstruction, Walter MacKenzie Health Sciences Centre, Edmonton, AB Canada; 3 Faculty of Medicine and Dentistry, Undergraduate Medical Education, University of Alberta, Edmonton, AB Canada; 4 Otolaryngology-Head and Neck Surgery Research Laboratory of Alberta, University of Alberta, Edmonton, AB Canada

## Abstract

**Background:**

Recent guidelines for the management of thyroid nodules incorporate mutation testing as an adjunct for surgical decision-making, however current tests are costly with limited accuracy. Droplet digital PCR (ddPCR) is an ultrasensitive method of nucleic acid detection that is particularly useful for identifying gene mutations. This study aimed to assess the analytic and clinical validity of RAS and BRAF ddPCR mutational testing as a diagnostic tool for thyroid fine needle aspirate biopsy (FNAB).

**Methods:**

Patients with thyroid nodules meeting indication for FNAB were prospectively enrolled from March 2015 to September 2017. In addition to clinical protocol, an additional FNAB was obtained for ddPCR. Optimized ddPCR probes were used to detect mutations including HRASG12 V, HRASQ61K, HRASQ61R, NRASQ61R, NRASQ61K and BRAFV600E. The diagnostic performance of *BRAF* and *RAS* mutations was assessed individually or in combination with Bethesda classification against final surgical pathology.

**Results:**

A total of 208 patients underwent FNAB and mutational testing with the following Bethesda cytologic classification: 26.9% non-diagnostic, 55.2% benign, 5.3% FLUS/AUS, 2.9% FN/SPN, 2.4% SFM and 7.2% malignant. Adequate RNA was obtained from 91.3% (190) FNABs from which mutations were identified in 21.1% of HRAS, 11.5% of NRAS and 7.4% of BRAF. Malignant cytology or BRAFV600E was 100% specific for malignancy. Combining cytology with ddPCR BRAF600E mutations testing increased the sensitivity of Bethesda classification from 41.7 to 75%. Combined BRAFV600E and Bethesda results had a positive predictive value (PPV) of 100% and negative predictive value (NPV) of 89.7% for thyroid malignancy in our cohort.

**Conclusions:**

DdPCR offers a novel and ultrasensitive method of detecting RAS and BRAF mutations from thyroid FNABs. BRAFV600E mutation testing by ddPCR may serve as a useful adjunct to increase sensitivity and specificity of thyroid FNAB.

**Electronic supplementary material:**

The online version of this article (10.1186/s40463-018-0299-2) contains supplementary material, which is available to authorized users.

## Background

The incidence of thyroid nodules may be as high as 70% in the adult population. Based on clinical and sonographic features, further diagnostic work-up is largely based on cytologic analysis of fine needle aspirate biopsy (FNAB). Unfortunately, up to 30% of FNABs are inconclusive and as a result of inaccurate pre-operative diagnosis, many patients with thyroid nodules undergo unnecessary surgery [1, 2]. Molecular analysis of thyroid FNABs has been shown to improve diagnostic accuracy [3]. Incorporating these findings, recent American and European guidelines support the use of mutation testing of genes associated with thyroid cancer (*BRAF, RAS, RET/PTC, PAX8/PPARG*) in order to improve surgical decision making [3, 4].

The most common mutation associated with thyroid cancer involves *BRAF* codon V600, followed by mutations in *RAS* [5]. The *BRAF* activating V600E mutation (BRAFV600E) is found in 29–83% of papillary thyroid cancers (PTC), and is associated with more aggressive disease [4, 6–8]. A number of *RAS* mutations have been associated with thyroid cancer, with variable diagnostic utility [5]. Mutations in codon 61 of *HRAS* and *NRAS* are thought to have the highest positive predictive value for malignancy (85–87%) [5, 9]. Data from The Cancer Genome Atlas demonstrates that alterations in *BRAF* and *RAS* enable molecular classification of PTC subtypes that is more representative of their differences in tumor biology than histopathologic classification [10]. Recent exploration of the mutational landscape of follicular thyroid cancers (FTCs) has suggested that perhaps well differentiated thyroid cancers could best be classified in three molecular subtypes: BRAF-like, RAS-like and Non-BRAF-Non-RAS [10]. Yet numerous genetic alterations have been identified as potential diagnostic markers for thyroid cancer, many of which are used in commercial tests with inconsistent clinical performance [4].

A major limitation of current molecular tests for thyroid cancer is these assays require large volumes of high quality RNA, often lacking from FNABs. This amount of genetic material is required for amplification of low copy mutations attributed with thyroid cancers. Recent advancements in nucleic acid detection using digital droplet PCR (ddPCR) can circumvent these limitations [11, 12]. DdPCR is a rapid and ultrasensitive method of detecting nucleic acid targets, shown to be particularly useful for the identification of mutant alleles in a variety of cancers [6, 12–14]. This technology has recently been employed for the rapid and accurate detection of BRAFV600E in colorectal cancer and melanoma [6, 15]. Given the precision of ddPCR for mutation detection, especially with nucleic acids of low abundance, it is an ideal molecular diagnostic tool for FNAB that has not yet been utilized for this purpose. We describe the first use of ddPCR for the detection of *RAS* and *BRAF* mutations in thyroid nodules.

## Methods

### Patients

Patients presenting to the University of Alberta Head and Neck Clinic for consultation regarding a thyroid nodule were prospectively recruited and consented for enrolment in this study from March 2015 to September 2017, in keeping with approved health research ethics board protocols (Pro00062302 and Pro00016426) . An ultrasound-guided fine needle aspirate biopsy (FNAB) was performed as standard of care for cytology, with an additional needle pass taken for ddPCR analysis immediately transferred to a 1.5 mL tube containing 200 ul RNA*later*™ (Thermofisher AM7021). FNA samples suspended in RNAlater were kept at room temperature < 24 h and at 4°C for < 7 days until processed for RNA extraction. Determination of mutation status by ddPCR was performed by MK, who was blinded to clinical and pathologic characteristics associated with FNAB samples. Decision to treat patients surgically followed 2015 American Thyroid Association (ATA) guidelines [3] and was not influenced by ddPCR mutation results.

### RNA extraction and cDNA synthesis

RNA was extracted using the RNeasy PlusMini Kit (Qiagen Cat No./ID: 79656). 550 ul of Buffer RLT, 40 mM DTT was added directly to the tube containing the FNA and the tube was vortexed extensively. The sample was loaded onto a QIAshredder (Qiagen Cat No./ID: 79656) and centrifuged at 8000 x g for 30 s at room temperature. The resulting flow through was loaded onto a gDNA Eliminator mini Spin Column and centrifuged 30 s at 8000 x g. An equal volume of 70% ethanol was added to the flow through, mixed by pipetting, and the mixture was transferred to an RNeasy Mini spin column and centrifuged for 15 s at 8000 x g. Following RNA binding, the Mini column was washed as per manufacturer’s instructions and the RNA was eluted with 50 ul RNase free H2O. RNA concentration was quantified using the Qubit RNA HS assay kit on a Qubit 2.0 fluorometer as per manufacturer’s instructions. The RNA was either stored at -80^o^ C or immediately used to carry out cDNA synthesis.

RNA (5–500 ng) was used to synthesize cDNA using the iScriptTM Reverse Transcription Supermix for RT-qPCR (BIO-RAD) as per the manufacturer’s protocol. Following the reaction, the cDNA was diluted with nuclease free H_2_O to a final concentration of 1 ng/ul (if initial RNA concentration was high enough) or, in some cases, 2 ng/ul. Newly synthesized cDNA was either stored at -20^o^ C or used directly for ddPCR.

### ddPCR reactions

Reactions were set up following the manufacturer’s protocols using 12 ul/reaction of 2× ddPCR Supermix for Probes (No dUTP), 1.2 ul/reaction of 20× mutant primers/probe (FAM BIO-RAD), 1.2 ul/reaction 20× wildtype primers/probe (HEX, BIO-RAD), 2.4 ul cDNA (at up to 2 ng/ul) and 7.2 ul H2O. ddPCR was carried out using the ddPCRTM Supermix for Probes (No dUTP) (BIO-RAD), the QX200TM Droplet Generator (catalog #186–4002 BIO-RAD), the QX200 Droplet Reader (catalog #186–4003 BIO-RAD) the C1000 TouchTM Thermal Cycler (catalog #185–1197 BIO-RAD) and the PX1TM PCR Plate Sealer (catalog #181-40well plate, mixed using a Mixmate Vortex Shaker (Eppendorf) and 20 ul of the reaction mixture was transferred to DG8TM Cartridge for QX200/QX100 Droplet Generator (catalog #186–4008 BIO-RAD) followed by 70 μl of Droplet Generation Oil for Probes (catalog #186–3005 BIO-RAD) into the oil wells, according to the QX200 Droplet Generator Instruction Manual (#10031907 BIO-RAD). Following droplet generation, 40 ul of the reaction was transferred to wells of a 96 well plate and the reactions were carried out in the thermocycler using the following parameters: Step 1) 95^o^ C for 10 min, Step 2) 94^o^ C for 30 s and 60^o^ C for 1 min (Step 2 repeat 39 times for a total of 40), Step 3) 98^o^ C for 10 min and Step 4) 4^o^ C infinite hold. All steps had a ramp rate of 3^o^ C/second. Following thermocycling the reactions were read in the QX200 Droplet Reader and the RNA targets were quantified using the QuantaSoftTM Software (BIO-RAD).

BIO-RAD proprietary ddPCR Primers and probes used were as follows: Unique Assay ID dHsaCP2000026 PrimePCR ddPCR Mutation Assay HRAS WT for p.G12 V Human, Unique Assay ID dHsaCP2000025 PrimePCR ddPCR Mutation Assay HRAS p.G12 V Human, Unique Assay ID dHsaCP2506815 PrimePCR ddPCR Mutation Assay HRAS WT for p.Q61K Human, Unique Assay ID dHsaCP2506814 PrimePCR ddPCR Mutation Assay HRAS p.Q61K Human, Unique Assay ID dHsaCP2500577 PrimePCR ddPCR Mutation Assay HRAS WT for p.Q61R Human, Unique Assay ID dHsaCP2500576 PrimePCR ddPCR Mutation Assay HRAS p.Q61R Human, Unique Assay ID dHsaCP2000068 PrimePCR ddPCR Mutation Assay NRAS WT for p.Q61K Human, Unique Assay ID dHsaCP2000067 PrimePCR ddPCR Mutation Assay NRAS p.Q61K Human, Unique Assay ID dHsaCP2000072 PrimePCR ddPCR Mutation Assay NRAS WT for p.Q61R Human, Unique Assay ID dHsaCP2000071 PrimePCR ddPCR Mutation Assay NRAS p.Q61R Human, Unique Assay ID dHsaCP2000028 PrimePCR ddPCR Mutation Assay BRAF WT for p.V600E Human, Unique Assay ID dHsaCP2000037 PrimePCR ddPCR Mutation Assay BRAF p.V600R Human. Determination of mutant versus wild type RAS and BRAF samples was based on the presence or absence of mutant droplets in the expected regions in two-dimensional data output plots determined using Quantasoft (Additional file 1: Figure S1). The first 98 collected FNA samples were repeated 2 or more times and demonstrated completely reproducible results for the detection of mutations.

### Statistics

Statistical calculations were completed using SPSS version 25 (IBM, Chicago, IL) and MedCalc 12.2 where appropriate. Bayesian statistics were used to calculate means, Pearson correlation and Loglinear regression. The performance of standard pathology (Bethesda classification) and ddPCR mutation profiling was estimated using Bayes theorem. Where appropriate, 95% confidence intervals were calculated using Clopper-Pearson for sensitivity and specificity, the Log method for positive likelihood ratios (PLR) and negative likelihood ratios (NLR) [16], and standard logit for positive predictive value (PPV) and negative predictive value (NPV) [17].

## Results

**Fig. 1:**
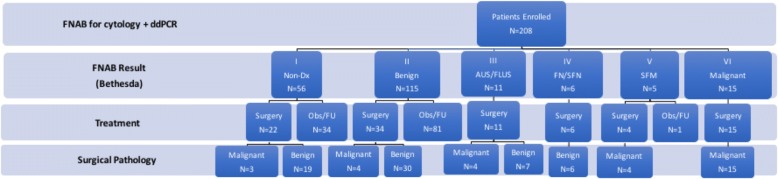
Flow diagram of patients included in this study

A total of 208 patients with thyroid nodules were prospectively enrolled for participation in this study. FNAB results from standard of care cytology yielded the following distribution in Bethesda classification: 26.9% (56) non-diagnostic, 55.2% (115) benign, 5.3% (11) AUS/FLUS, 2.9% (6) FN/SFN, 2.4% (5) SFM and 7.2% (15) malignant (Fig. 1). Based on clinical, sonographic and cytologic characteristics, thyroid surgery was performed on 44.2% (92) of patients in this cohort (Table 1 and Fig. 1). Of patients who were classified as Bethesda III-VI (17.8%), only 1 patient, who was Bethesda V, did not receive surgical intervention. All patients with Bethesda V or VI (9.1%) were found to have papillary thyroid cancer (PTC) on final surgical pathology (Fig. 1 and Additional file 2: Table S1). Seven patients (12.5%) had thyroid cancer (6 PTC, 1 FTC) with pre-operative cytology that was benign or non-diagnostic. Four patients (36.3%) had thyroid cancer (2 PTC, 2 FTC) with pre-operative cytology classified as AUS/FLUS.

**Table 1:** Clinicopathologic characteristics of patients with thyroid nodules enrolled in this study

Variable	All (%) *N* = 208	Bethesda Categories (%)					
		I (non-dx) *N* = 56	II (benign) *N* = 115	III (AUS/FLUS) *N* = 11	IV (FN/SFN) *N* = 6	V (SFM) *N* = 5	IV (malignant) *N* = 15
Age							
Mean	54.8	55.6	54.2	51.2	67.3	52.8	55.5
< 45	22.1	19.6	24.3	27.2	16.7	0	20.0
> 45	77.9	81.8	75.6	72.3	83.3	100.0	80.0
Sex (female)	67.8	23.6	46.2	81.8	50.0	80.0	66.7
Nodule size							
1.0–3.9 cm	90.4	100	86.1	100	66.7	100	86.7
≥4.0 cm	9.6	0	13.9	0	33.3	0	13.3
Sonographic Risk Features							
High	11.3	3.8	8.4	11.1	0	40.0	61.5
Intermediate	25.2	19.2	28.4	44.4	16.7	40.0	7.7
Low	39.7	48.1	38.5	22.2	83.3	0	23.1
Very low	18.0	21.2	18.3	22.2	0	20.0	7.7
Benign	5.6	7.7	6.4	0	0	0	0
Surgery Performed (%)	92 (44.2)	22 (39.2)	34 (29.6)	11 (100)	6 (100)	4 (80)	15 (100)

*AUS/FLUS* atypia of uncertain significance/follicular lesion of undetermined significance, *FN/SFN* follicular neoplasm/suspicious for follicular neoplasm, *SFM* suspicious for malignancy

An additional FNA sample for ddPCR analysis was obtained for all patients enrolled in this study. Following RNA extraction, mean concentration of nucleic acid obtained per sample was 11.6 μg/ml (3.48 μg total). Nineteen (9.1%) FNA samples did not have adequate amounts of RNA (< 0.001 μg) for ddPCR analysis (Table 2). Of patients who had non-diagnostic pathology (*N* = 56, 26.9%), 91% (51/56) of samples contained sufficient high-quality RNA for ddPCR. Overall, HRASQ61R was the most common mutation identified (19.7%), followed by HRASG12 V (17.3%), NRASQ61R (8.2%), HRASQ61K (6.7%), BRAFV600E (6.7%) and NRASQ61K (1.9%). All patients with SFM or malignant cytology (Bethesda V or VI) harbored at least one mutation in *RAS* or *BRAF*.

**Table 2:** Distribution of RAS and BRAF mutations identified by ddPCR according to Bethesda classification

Bethesda	Low RNA	HRAS G12 V	HRAS Q61R	HRAS Q61K	NRAS Q61R	NRAS Q61K	BRAF V600E
1-Non Dx *N* = 56 (26.9)	5	9	6	6	2	1	1
2-Benign *N* = 115 (55.2)	11	20	28	6	11	2	1
3-FLUS/AUS *N* = 11 (5.3)	2	2	2	2	1	1	1
4-FN/SFN *N* = 6 (2.9)	0	1	1	0	1	0	0
5-SFM *N* = 5 (2.4)	1	1	1	0	1	0	2
6-Malignant *N* = 15 (7.2)	0	3	3	0	2	0	9
Total (%)	19 (9.1)	36 (17.3)	41 (19.7)	14 (6.7)	18 (8.2)	4 (1.9)	14 (6.7)

Low RNA column indicates FNAB samples with RNA/nucleic acid < 1 ng, not used for ddPCR analysis

*Dx* diagnostic, *AUS/FLUS* atypia of uncertain significance/follicular lesion of undetermined significance, *FN/SFN* follicular neoplasm/suspicious for follicular neoplasm, *SFM* suspicious for malignancy

In patients who received thyroid surgery, a higher percentage of BRAFV600E mutations was found compared to the entire cohort (15.2% vs 6.7%). All patients with a BRAFV600E mutation were found to have PTC on final pathology (Table 3 and Additional file 2: Table S2). Of these patients, 36% (5/14) also harbored a HRAS mutation (1 HRASG12 V, 4 HRASQR1R). In patients with FTC, 2 *RAS* mutations and no *BRAF* mutations were identified (Additional file 2: Table S2). A lower number of *RAS* mutations was found in patients with thyroid cancer compared to benign pathology (19.5% vs 50.0%). Only 3.2% of patients who received surgery did not have adequate RNA for ddPCR, whereas 23.9% of patients had non-diagnostic cytology (Bethesda I).

**Table 3:** Distribution of pre-operative fine needle aspirate cytology and ddPCR mutation results in surgical specimen

	Surgical Pathology		
	Benign (%)	Malignant (%)	Total (%)
Cytology (Bethesday)			
I –Non- diagnostic	19 (20.7)	3 (3.2)	22 (23.9)
II - Benign	30 (32.6)	4 (4.3)	34 (40.0)
III – AUS/FLUS	7 (7.6)	4 (4.3)	11 (12.0)
IV – FN/SFN	6 (6.5)	0	6 (6.5)
V - SFM	0	4 (4.3)	4 (6.5)
VI- Malignant	0	15 (16.3)	15 (16.3)
Mutations			
Low RNA	2 (2.2)	1 (1.1)	3 (3.2)
HRASG12 V	14 (15.2)	6 (6.5)	20 (21.7)
HRASQ61R	14 (15.2)	5 (5.4)	19 (20.7)
HRASQ61K	8 (8.7)	1 (1.1)	9 (9.8)
NRASQ61R	6 (6.5)	5 (5.4)	11 (12.0)
NRASQ61K	3 (3.2)	0	3 (3.2)
BRAFV600E	0	14 (15.2)	14 (15.2)

Low RNA column indicates FNAB samples with RNA/nucleic acid < 1 ng, not used for ddPCR analysis

*AUS/FLUS* atypia of uncertain significance/follicular lesion of undetermined significance, *FN/SFN* follicular neoplasm/suspicious for follicular neoplasm, *SFM* suspicious for malignancy

**Fig. 2:**
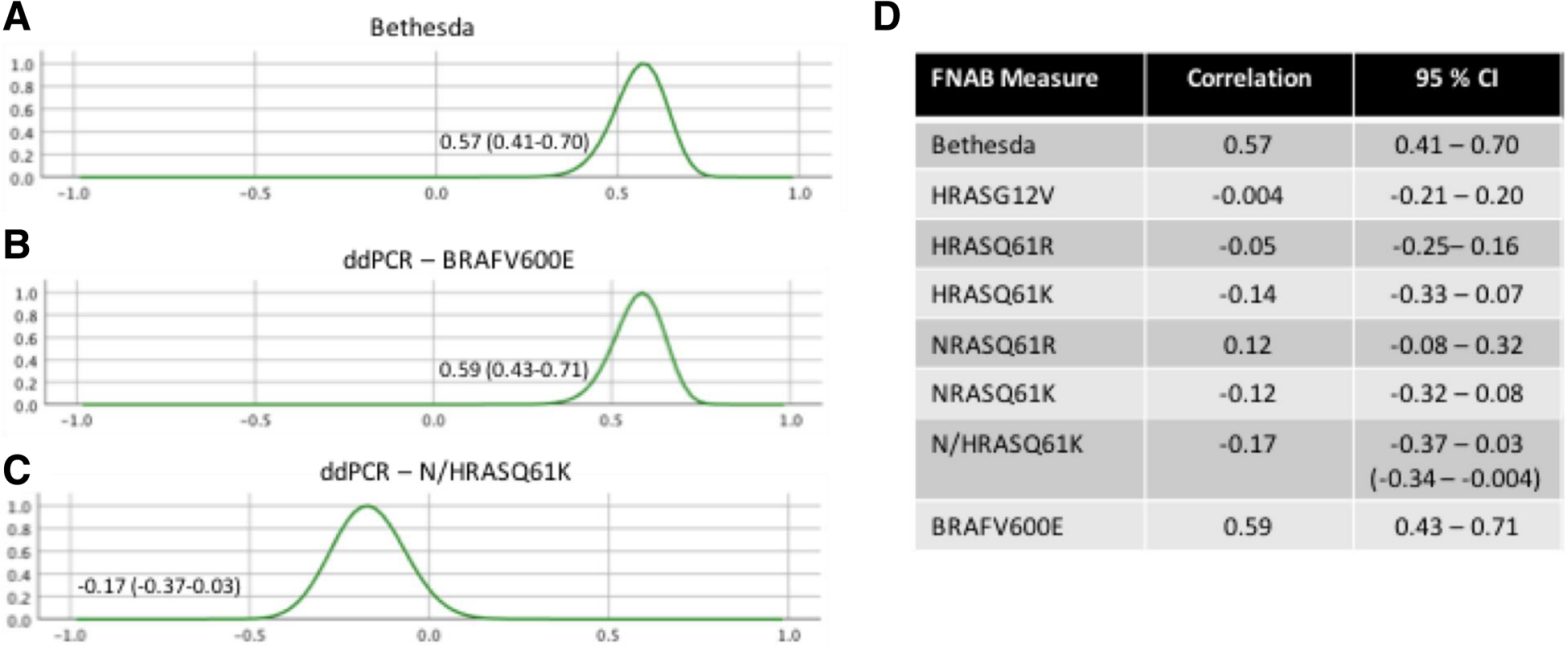
Correlation of Bethesda classification and ddPCR with surgical pathology. Correlation between diagnosis of thyroid cancer on surgical pathology and pre-surgical FNAB and **a**) Bethesda classification, **b**) ddPCR detection of BRAFV600E and **c**) N/HRASQ61K. **d** Pearson correlation values final surgical pathology diagnosis of thyroid cancer and Bethesda cytology, in addition to ddPCR mutations in RAS or BRAF. N/KRASQ61K does not cross 0 at 90% credible interval shown in brackets

Correlative analysis between pre-operative Bethesda classification, RAS/BRAF mutations and final surgical pathology was performed (Fig. 2). The Bethesda classification showed statistically significant correlation with malignant vs benign pathology (0.57, 95% CI 0.41–0.70). BRAFV600E mutation had a slightly higher but similar correlation with surgical pathology results (0.59, 95% CI 0.43–0.71). Individual *RAS* mutations had no significant correlation with pathology, however, combined N/HRASQ61K was negatively correlated with thyroid cancer (− 0.17, 95% CI -0.37 - 0.03, 90% CI -0.34 - -0.004).

The diagnostic performance of Bethesda classification and RAS/BRAF mutation testing is shown in Table 4. Bethesda *V*/VI was 100% specific and 41.7% sensitive for thyroid cancer. When including all Bethesda categories that could recommend surgical intervention (Bethesda III-VI), specificity is lowered to 70.7% for a 3% improvement in sensitivity (44.7%). BRAFV600E testing provided 100% specificity and 50% sensitivity for the diagnosis of thyroid cancer. Combining the Bethesda system with BRAFV600E, higher sensitivity is achieved (75%) while maintaining 100% specificity. The addition of H/NRASQ61K mutations results in minimal increase in sensitivity (77.8%) and decrease in specificity (98.4%).

**Table 4:** Comparative diagnostic performance of pre-operative standard cytology and ddPCR mutation testing

MEASURE	Bethesda III-VI	Bethesda V/VI	BRAFV600E	BRAFV600E + BETHESDA *V*/VI^b^	BRAFV600E + H/NRASQ61K + Bethesda *V*/VI
Sensitivity	44.7 (30.2–59.9)	41.7 (27.6–56.8)	50 (30.7–69.4)	75.0 (55.1–89.3)	77.8 (57.7–91.4)
Specificity	70.7 (54.5–83.9)	100 (92.6–100)	100 (94.1–100)	100 (94.1–100)	98.4 (91.3–100)
PPV^a^	63.6 (49.7–75.6)	100	100	100	95.5 (74.8–99.3)
NPV^a^	52.7 (44.6–60.6)	60 (54.1–65.6)	81.3 (75.1–86.3)	89.7 (82.1–94.3)	91 (83.4–95.4)
PLR	1.53 (0.86–2.71)	–	–	–	48.2 (6.8–340.5)
NLR	0.78 (0.6–1.1)	0.6 (0.5–0.8)	0.5 (0.4–0.7)	0.25 (0.1–0.5)	0.2 (0.11–0.5)

*NLR* negative likelihood ratio, *NPV* negative predictive value, *PLR* positive likelihood ratio, *PPV* positive predictive value

^a^Because the sample sizes in disease positive and disease negative groups may not reflect the true population prevalence of the disease, PPV and NPV may be inaccurate [9]. 95% confidence interval is shown in brackets where appropriate

^b^Combined BRAF and Bethesda V/VI classifies test as positive if BRAFV600E and/or Bethesda *V*/VI is present

## Discussion

We describe the first use of ddPCR for the identification of *RAS* and *BRAF* mutations from thyroid FNAB samples. With the addition of a single needle sample taken as part of standard of care FNAB, adequate material for ddPCR mutation analysis was obtained in > 90% of cases. In contrast, 26.9% of FNAB were cytologically non-diagnostic. Consistent with other studies [18], the identification of BRAFV600E alone in our cohort was 100% specific for thyroid cancer, with sensitivity comparable to standard cytology. By combining the Bethesda system with BRAFV600E ddPCR testing, the sensitivity of FNAB diagnosis markedly increased while maintaining high specificity. As shown by our group and others, ddPCR analysis can provide rapid results (< 24 h) that are highly reproducible and accurate, requires minimal nucleic acid sample and can be performed at lower cost than standard pathology [6, 11, 13, 14]. BRAFV600E testing by ddPCR circumvents limitations of other currently available molecular tests and therefore has the potential to be of clinical utility. Recent studies in melanoma and colorectal cancer have demonstrated the clinical potential of BRAFV600E testing by ddPCR as a highly accurate and low-cost molecular test [6, 15].

This study aimed to identify somatic mutations most commonly found in well differentiated thyroid cancers, which includes *BRAF* and *RAS*. Using a PCR based approach in a large cohort, Moses et al. suggested *BRAF* and *RAS* mutation testing of FNAB could improve the rate of definitive surgical management [2]. An independent study suggested improved diagnostic accuracy of FNABs could be obtained by molecular profiling of *N/HRAS* and *BRAF* [18]. A more recent study evaluated use of next generation sequencing (NGS) analysis of thyroid nodules compared to surgical pathology in 63 patients (10/63 malignant) [19]. Consistent with our data, *RAS* mutations were commonly found but had low PPV (9%), whereas BRAFV600E had 100% PPV for malignancy. However, given the amount of nucleic acid required and cost of NGS, ddPCR analysis may be a preferred method for FNAB [6].

*RAS* mutations are the second most common genetic alteration in thyroid cancer, yet their role remains unclear for clinical management. A recent meta-analysis pooling 1025 patients found that *RAS* mutations were 34.3% sensitive and 93.5% specific for the detection of malignancy in indeterminate thyroid FNABs [5]. This study only included Bethesda III-V lesions, excluding thyroid adenomas (Bethesda II, benign) known to harbor *RAS* mutations in 20% to 40% of cases [20]. A literature review of 36 molecular markers used to increase the diagnostic accuracy of thyroid FNAB found that *RAS* mutations had the lowest sensitivity among these [21]. The most recent European Thyroid Association Guidelines state that *RAS* mutations are associated with a higher risk of malignancy but should not be used to dictate more aggressive surgical intervention. Our data are consistent with the literature, identifying a high number of *RAS* mutations in benign disease, with low correlation to malignancy. In our surgical cohort only 10% of malignancies were FTCs, more commonly associated with *RAS* mutations, with the remaining 90% consisting of PTCs, known to have a low association with *RAS*. Given the higher number of follicular adenomas vs carcinomas in our cohort, *RAS* mutations were expectedly higher in the benign vs malignant group. It is possible that in a larger cohort of indeterminate nodules (excluding benign), *RAS* mutation testing by ddPCR could be of predictive value as suggested by others [5].

The Bethesda system for reporting FNA cytology is currently the most widely adopted classification scheme [4]. The 2017 revision confirms this system to be robust, maintaining *status quo* in the six diagnostic categories [22]. For lesions in category V and VI (suspicious for malignancy and malignant), high specificity for malignancy warrants surgical intervention in most cases. Nevertheless, up to 30% of cases may be classified in an “indeterminate” category (III/IV) that requires diagnostic surgery. An estimated 70% to 80% of these cases will be found to have benign pathology following surgery. This is a diagnostic limitation that is associated with a tremendous burden on healthcare utilization and costs. In our study cohort, 76.5% of patients who had indeterminate (III/IV) cytology were found to have benign pathology. In addition, malignant pathology was found in 11.7% of patients who were classified as having benign disease. Although ddPCR results were not used for treatment decisions in this study, our results suggest that BRAFV600E mutation testing could have triaged patients to expedite appropriate surgical care.

In response to the increasing incidence of thyroid nodules and the significant, potentially avoidable, healthcare costs associated with diagnostic thyroid surgery a number of commercial molecular tests have been developed. Among the most commonly utilized tests include the Afirma Gene Expression Classifier (GEC) ($4875, $475 for *BRAF* only), ThyGenX ($1675), ThyraMIR ($3300) and ThyroSeq ($3200) [23]. The Afirma GEC and ThyroSeq have high NPV but low PPV, whereas ThyGenX is thought to have high PPV with low NPV [19, 24]. ThyMIR (when combined with ThyGenX) may provide good NPV and PPV but validation data is limited [23]. The 2015 ATA guidelines suggest the use of molecular testing in specific instances for nodules with Bethesda class III-V, however the level of quality evidence is currently weak to moderate [3, 9]. In terms of heathcare savings, it has been suggested that at a cost of $3200/test, $1453 could be saved on total cost of care. This calculation is based on an assumed number of diagnostic surgeries avoided [4, 23]. In an economic model based on patients with a single indeterminate FNA, it has been estimated that healthcare savings could be obtained if the cost of molecular testing is less than $870/test [25]. Data from our study suggests combining ddPCR BRAFV600E testing with Bethesda cytology results can achieve high PPV (100%) and NPV (89.7%), comparable to other commercially available tests [9, 23]. The estimated cost for ddPCR of BRAFV600E is $20.45/FNAB [14] in addition to standard of care cytologic analysis (Bethesda), which is varies between health care regions. For certain thyroid nodules, ddPCR testing combined with Bethesda grading may be economically advantageous over currently available commercial assays, however further analysis in the context of a clinical utility study would be required.

With the goal of improving FNA diagnostics, research efforts have been predominantly focused on the molecular classification of indeterminate cytology, with little attention paid to the resolution of non-diagnostic (Bethesda I) results [4]. The rate of non-diagnostic FNAB ranges from 2 to 36%, depending on several factors including the sonographic characteristics of a nodule, the technique and experience of the physician obtaining the biopsy, and the experience of the cytopathologist [1, 26, 27]. The rate of non-diagnostic FNAB in our study cohort was 26.9%, similar to an earlier study with a cohort from the same institution (23%) [1]. RNA of sufficient quality was obtained in 91% of non-diagnostic specimen, of which one BRAFV600E mutation was identified in one PTC. Given the known ultrasensitive properties of ddPCR, this may be a useful clinical tool to triage non-diagnostic cytology.

As the first study to investigate the use of ddPCR mutation testing of FNABs, a number of limitations require consideration to address the potential clinical utility of this this test. This is a single centre experience in a tertiary referral clinic consisting of head and neck oncologic surgeons, therefore creating an inherent bias toward patients who are more likely to require surgical intervention. A single centre study is limited in the generalizability of results given that differences in the diagnostic yield of FNAB cytology and molecular testing are known to vary between centres [28]. The sensitivity and specificity calculated in our cohort may be affected by disease prevalence, however unlike PPV and NPV these measures of test performance are most often not affected by prevalence [29]. The performance of ddPCR mutation analysis from FNAB is measured against surgical pathology, however, ddPCR was not done on surgical pathology specimen for comparison. This places some limitation on our understanding of the true performance of ddPCR from an FNA especially in genetically heterogenous tumors where results could be dependent on where the biopsy is being taken. Comparison of pre- and post-surgical samples would be required to further define the analytic validity of ddPCR mutation testing in FNAB. Although this study was conducted over the course of 30 months, the follow-up time may not have been adequate to determine if some patients with *RAS* mutations go on to develop malignancy. In addition, only somatic mutations thought to provide the highest-yield information were included in this study. Given that ddPCR has been shown to be superior to other techniques for the identification of point mutations, primers and/or probes can be designed to develop a multiplex assay that uncovers other mutations as has been done by others. Re-analysis of FNAB samples processed for ddPCR with a larger panel of mutations and long-term follow-up may provide further insight. A larger, multi-institutional study would be an important step in assessing the clinical utility of ddPCR mutation testing as a diagnostic tool for FNAB.

## Conclusions

DdPCR offers a novel and ultrasensitive method of detecting *RAS* and *BRAF* mutations from thyroid FNABs. BRAFV600E mutation testing by ddPCR may serve as a useful adjunct to increase sensitivity and specificity thyroid FNAB. Further studies are required to determine the diagnostic utility of ddPCR mutational testing of thyroid FNABs.

## Additional files

Additional file 1: **Figure S1.** Identification of BRAF and RAS mutations by ddPCR. Two-dimensional data outputs showing an example of a A) BRAF^V600E^ positive FNAB sample demonstrating BRAF^V600E^mutant (FAM+, blue) and BRAF^WT^ (HEX+, green) copies and B) a FNAB sample harboring a NRASQ61K (FAM+, blue, 44 copies), shown compared to NRAS^WT^ (HEX+, green). Only samples containing droplets with clear separation from the baseline and directly vertical to the baseline were considered as positive for the mutation in question. Distribution of mutant probes was as expected, correlating with BIO-RAD data from proprietary assays. Samples only containing droplets at a 45^o^ angle to the baseline (suggestive of containing both mutant and wildtype) were considered as false positives in this study. ddPCR, droplet digital PCR; HEX, hexachloro-fluorescein; FAM, 6-carboxyfluorescein; FNAB, fine needle aspirate biopsy. (DOCX 1031 kb)

Additional file 2: **Table S1.** Distribution of pre-operative fine needle aspirate cytology results in surgical specimen. **Table S2.** ddPCR mutational profile according to final surgical pathology. (DOCX 18 kb)

## Abbreviations

ATA: American Thyroid Association
AUS/FLUS: Atypia of uncertain significance/follicular lesion of undetermined significance
BRAFV600E: BRAF valine to glutamic acid mutation at residue 600
cDNA: complementary deoxyribonucleic acid
ddPCR: droplet digital polymerase chain reaction
DNA: Deoxyribonucleic acid
DTT: 1,4-dithiothreitol
FAM: 6-carboxyfluorescein
FN/SFN: Follicular neoplasm/suspicious for follicular neoplasm
FTC: Follicular thyroid carcinoma
HEX: Hexachloro-fluorescein
miFTC: minimally invasive follicular thyroid carcinoma
NGS: Next generation sequencing
NLR: Negative likelihood ratio
NPV: Negative predictive value
PLR: Positive likelihood ratio
PPV: Positive predictive value
PTC: Papillary thyroid cancer
RNA: Ribonucleic acid
SFM: Suspicious for malignant cells
WT: Wild type
