# Supplementary figures and images for: Analytic and clinical validity of thyroid nodule mutational profiling using droplet digital polymerase chain reaction
Source: J Otolaryngol Head Neck Surg. 2018 Sep 24;47:60. doi: 10.1186/s40463-018-0299-2 (PMC6154415; doi:10.1186/s40463-018-0299-2)

**
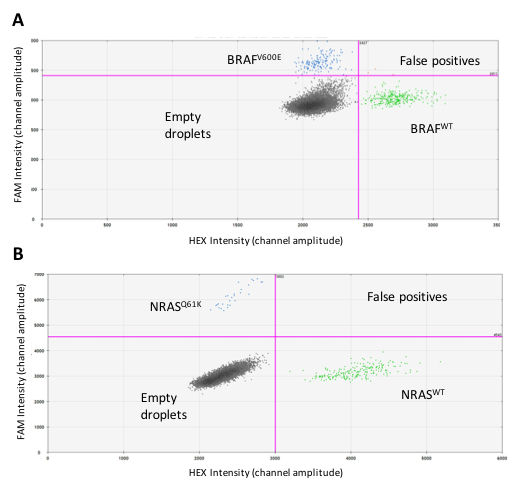
**

Supplement: Supplementary file 1 — Figure S1. Identification of BRAF and RAS mutations by ddPCR. Two-dimensional data outputs showing an example of a A) BRAFV600E positive FNAB sample demonstrating BRAFV600Emutant (FAM+, blue) and BRAFWT (HEX+, green) copies and B) a FNAB sample harboring a NRASQ61K (FAM+, blue, 44 copies), shown compared to NRASWT (HEX+, green). Only samples containing droplets with clear separation from the baseline and directly vertical to the baseline were considered as positive for the mutation in question. Distribution of mutant probes was as expected, correlating with BIO-RAD data from proprietary assays. Samples only containing droplets at a 45o angle to the baseline (suggestive of containing both mutant and wildtype) were considered as false positives in this study. ddPCR, droplet digital PCR; HEX, hexachloro-fluorescein; FAM, 6-carboxyfluorescein; FNAB, fine needle aspirate biopsy. (DOCX 1031 kb) [file 40463_2018_299_MOESM1_ESM.docx]
