# Supplementary material for: Analytic and clinical validity of thyroid nodule mutational profiling using droplet digital polymerase chain reaction
Source: J Otolaryngol Head Neck Surg. 2018 Sep 24;47:60. doi: 10.1186/s40463-018-0299-2 (PMC6154415; doi:10.1186/s40463-018-0299-2)
Supplement: Supplementary file 2 — Table S1. Distribution of pre-operative fine needle aspirate cytology results in surgical specimen. Table S2. ddPCR mutational profile according to final surgical pathology. (DOCX 18 kb) [file 40463_2018_299_MOESM2_ESM.docx]

**Additional file 2**

**Table S1.** Distribution of pre-operative fine needle aspirate cytology results in surgical specimen.

| **Pathology** | **I**  (non-dx) | **II**  (benign) | **III**  **(**AUS/FLUS) | **IV**  (FN/SFN) | **V**  (SFM) | **IV**  (malignant) |
| --- | --- | --- | --- | --- | --- | --- |
| **Benign**  **Adenomatoid**  **Hyperplasia**  **Thyroiditis**  **Follicular adenoma**  **Multinodular goitre** | 19  3  5  0  2  9 | 30  11  6  4  4  6 | 7  2  1  1  3  0 | 6  1  2  0  3  0 | 0  0  0  0  0  0 | 0  0  0  0  0  0 |
| **Malignant**  **PTC**  **FTC** | 3  3  0 | 4  3  1 | 4  2  2 | 0  0  0 | 4  4  0 | 15  15  0 |
| **Total (%)** | **22** | **34** | **11** | **6** | **4** | **15** |

FTC, follicular thyroid carcinoma; PTC, papillary thyroid cancer

**Table S2.** ddPCR mutational profile according to final surgical pathology

| **Pathology** | **Low RNA** | **HRAS**  **G12V** | **HRAS**  **Q61R** | **HRAS**  **Q61K** | **NRAS**  **Q61R** | **NRAS**  **Q61K** | **BRAF**  **V600E** |
| --- | --- | --- | --- | --- | --- | --- | --- |
| **Benign**  **Adenomatoid**  **Hyperplasia**  **Thyroiditis**  **Follicular adenoma**  **Multinodular goitre** | 2  1  1  0  0  0 | 14  6  6  0  2  1 | 14  4  3  3  1  4 | 8  4  1  0  2  1 | 6  4  0  0  2  0 | 3  1  0  1  1  0 | 0  0  0  0  0  0 |
| **Malignant**  **PTC**  **FTC** | 1  1  0 | 6  5  1 | 5  5  0 | 1  1  0 | 5  4  1 | 0  0  0 | 14  14  0 |
| **Total (%)** | **3** | **20** | **19** | **9** | **11** | **3** | **14** |

FTC, follicular thyroid carcinoma; PTC, papillary thyroid cancer
